# Identifying Caregiving Youth and Associated Mental Health Concerns in a Medical Clinic Setting

**DOI:** 10.3390/healthcare13030255

**Published:** 2025-01-28

**Authors:** Elizabeth R. Pulgaron, Gabriella Llano, Gabriela Guevara, Tara Kenworthy LaMarca, Gwen Wurm, Lisa Gwynn, Connie Siskowski, Julia Belkowitz

**Affiliations:** 1 Miller School of Medicine, University of Miami Miller, Miami, FL 33136, USA; gxg598@miami.edu (G.G.); tlk38@miami.edu (T.K.L.); gwurm@med.miami.edu (G.W.); lgwynn@med.miami.edu (L.G.); 2 Holtz Children’s Hospital, Miami, FL 33136, USA; gabriella.llano@jhsmiami.org; 3 American Association of Caregiving Youth, Boca Raton, FL 33487, USA; connie@aacy.org

**Keywords:** caregiving youth, young carers, young caregivers, mental health

## Abstract

Background/ Objectives: Despite the high estimated prevalence and the documented impact of caregiving on children, there is no systematic process to identify or study caregiving youth in the healthcare setting. The aim of this study was to pilot screening in school-based clinics to identify caregiving youth and their associated mental health outcomes. Methods: From March 2021 to March 2022, ninth- to twelfth-grade students were surveyed regarding caregiving and validated mental health screeners during intake at three Title 1 school-based health clinics in Miami, FL. Results: Thirty-nine percent of participants self-identified as caregivers. The most common caregiving tasks were cleaning (n = 20, 50%), keeping company (n = 19, 48%), shopping/cooking (n = 14, 35%), dressing (n = 13, 33%), mobility support (n = 12, 30%), and medical support (n = 11, 28%). Compared to their non-caregiving counterparts, caregiving youth had higher scores on mental health screeners, and caregivers were more likely to endorse clinically significant levels of depression (*p* = 0.050). Conclusions: Screening in the healthcare system was effective at identifying caregiving youth in school-based clinics whose mental health may be impacted by caregiving responsibilities. Pediatricians should actively screen for both caregiving and mental health concerns. Future studies are needed to ensure the caregiving screening tool is reliable and valid for broad-scale provider use.

## 1. Introduction

Caregiving youth are “young people ages 18 and under who provide significant assistance or care to a family member who has a chronic illness, disability, mental health condition, and/or frailty due to aging [1]”. The first and only national study in the United States (2005) cited at least 1.3 million children aged 8–18 years in this role [2]. In 2020, the National Alliance for Caregiving/AARP acknowledged the existence of caregiving youth in its “Caregiving in the US” report and estimated that 5.4 million children and adolescents under 18 are providing care [3]. This is likely an underestimate as only children living with adult caregivers were studied, and therefore single parent/grandparent or other households in which a child is the only caregiver were excluded. In Florida, 16% of high school students reported caregiving at least once per week, and surveys of middle school children in our South Florida community showed high rates of caregiving [4,5].

Caregiving responsibilities range from direct healthcare to support with household tasks and are demanding, complex, and often long-lasting, regardless of the caregiver’s age [5,6,7]. Their responsibilities are demanding and often long-lasting and can be associated with emotional, social, and even physical effects. The Stress Process Model [8] is a framework used to explain caregiver stress in adults and has been applied to youth caregivers in previous studies [9]. The four primary domains of the model include background factors of caregiving youth (i.e., demographics, time spent caregiving), primary stressors of caregiving (i.e., caregiving tasks), secondary role strains (i.e., grades, conflict with caretakers), and outcomes (i.e., mental health). Mediators such as coping and social support are also included in the model. In recognition of both the potential burden and societal impact of family caregivers, both the Centers for Disease Control and Prevention (CDC) and the National Institutes of Health emphasize caregiving for their constituents, and the CDC formally recognizes caregiving as a public health priority [10,11]. However, neither organization formally acknowledges that children also take on the role of family caregivers.

Caregivers are known to play various and oftentimes changing roles that can have effects on their health and wellness. While there may be positive impacts of caregiving such as increased coping skills, pride, and maturity, it is well established that caregiving youth are at risk for poor mental health outcomes [12,13]. Their education may also be negatively impacted, with 22% of young adults who dropped out of school reporting doing so to care for a family member [14]. Decades of research from European countries and available studies from the United States have demonstrated that caregiving youth (known as young carers across Europe) are at increased risk for anxiety, depression, difficulties sleeping, and poor academic performance and may feel “invisible” in their schools and communities [4,6,13,15,16]. Additionally, the COVID-19 pandemic and resultant lockdown had great effects on youth. Studies examining the impact of the pandemic’s impact on the mental health of children and adolescents found associated psychological distress including symptoms of depression, anxiety, stress, isolation, and anger [17,18]. For caregiving youth, the COVID-19 pandemic created new pressures such as limited access to respite and support from community resources and mentors outside the home secondary to school closures [19].

Despite the high estimated prevalence both nationally and in our local community and the documented impact of caregiving on children, there is no systematic process to identify or study caregiving youth in the healthcare setting nor recommendations to do so in current practice guidelines such as the American Academy of Pediatrics’ Bright Futures Guidelines for Health Supervision [20]. In order to explore opportunities to identify caregiving youth in a clinical setting, the University of Miami School Health Clinics partnered with the American Association of Caregiving Youth (AACY) [1]. AACY is the only organization in the United States solely dedicated to supporting caregiving youth regardless of the diagnoses of the care receiver. Working in partnership with The School District of Palm Beach County in Florida, the AACY’s Caregiving Youth Project (CYP) screens sixth graders in participating middle schools to identify those with caregiving responsibilities and invite those within the top three of five Levels of Responsibility (LOR) to receive, with parental consent, supportive services. The LOR is a weighted index based on the types of activities the youth performs and the weekly time spent doing so. The aim of this study was to pilot screening in school-based clinics to identify caregiving youth and their associated mental health outcomes. A retrospective chart review was conducted to evaluate the findings from the administered surveys.

## 2. Materials and Methods

### 2.1. Participants

The screening sample comprised ninth through twelfth-grade students (n = 102) belonging to three Title I high schools in a Southeastern urban area. The majority of students (95–97% during the 2021–2022 school year) identified as Black or Hispanic [21]. Between 84% and 90% of students were eligible for free or reduced lunch, which is a proxy for socioeconomic and educational disadvantage [22]. All participants provided consent to the school’s school-based health center (SBHC), which offers health services free of charge to all students. This health center is connected to a larger network of SBHCs that offer services such as annual well-child visits, sick visits, vaccinations, and mental health services.

### 2.2. Materials

#### Mental Health Screeners

Five psychosocial measures were administered as part of the post-pandemic screening initiative to assess symptoms of depression, anxiety, and COVID-related worries. All measures were administered via a secure online survey system, Qualtrics, so that responses could be entered and automatically scored digitally. Demographic data were also collected, including students’ gender and age.

Patient Health Questionnaire (PHQ)-8. The PHQ-8 is a validated eight-item self-report measure derived from the diagnostic criteria for Major Depressive Disorder (MDD). Items are scored based on symptom frequency over the preceding two weeks from 0 (not at all) to 3 (nearly every day). The PHQ-8 is derived from a nine-item measure, the PHQ-9, but eliminates a question about suicidality. The PHQ-8 has been deemed appropriate and valid, particularly for large screening initiatives in which referral to appropriate services for suicidal patients may be a barrier [23]. A score of 10 or above is the commonly used cut-point [24], indicating moderate depressive symptoms, which was applied in this clinical sample. The PHQ-9 was found to have good sensitivity and specificity for detecting depression in adolescents [25], and the PHQ-8 reliably measured depression in youth [26].

Generalized Anxiety Disorder (GAD)-7. The GAD-7 is a validated seven-item self-report measure of symptoms of GAD, linked with GAD symptoms from the DSM-IV, which has shown good sensitivity and specificity for adolescents [27]. Scoring parallels the PHQ-8, with a scale from 0 to 3 and a score of 10 as the common cut point for this measure as well, representing moderate anxiety symptoms.

Pediatric Symptom Checklist (PSC)-17. The PSC-17 is a reliable and validated brief measure of children’s psychosocial functioning that is commonly used in primary care settings and research studies [28]. Its 17 items correspond with three subscales: Internalizing, Externalizing, and Attention. Cutoff scores are >15 for Total, >5 for Internalizing, >7 for Attention, and >7 for Externalizing. Scores above the cut points indicate significant impairments and risk for a behavioral health disorder.

Obsession with COVID-19 Scale (OCS). The OCS is a validated 4-item self-report measure of persistent and disturbed thinking about COVID-19 [29]. Items are rated on a 5-point time-anchored scale (0 = not at all, 4 = nearly every day over the past 2 weeks) with a cut score of 7 indicating too much COVID-19 thinking. This measure was validated for adults and has not yet been applied to adolescents.

Coronavirus Anxiety Scale (CAS). The CAS is a validated 5-item self-report measure of anxiety associated with COVID-19 [30]. Scoring options parallel the OCS’s 5-point scale, but a cutoff score of 9 indicates dysfunctional anxiety. This measure was also validated for adults and has not yet been applied to adolescents.

In addition to the mental health screeners, questions regarding caregiving responsibilities were administered. As there is no established screening tool to identify caregiving youth, questions from AACY’s school-based screeners were adapted for use. The survey consisted of the following items: (1) Do you provide care for someone in your family or household who is chronically ill (lasts 3 months or more), elderly, or disabled with activities they would have difficulty doing on their own? (multiple-choice response) (2) The person(s) in need of my help is my: (multiple-choice response) (3) What are the ways that you help the person(s) in need of care? (multiple-choice response with options related to activities of daily living) (4) Which other helping things do you also do? (multiple-choice response with options related to instrumental activities of daily living) (5) During school days, how many hours per day do you spend on your caring responsibilities? (multiple-choice response) (6) During the weekend, how many hours per day do you spend on your caring responsibilities? (multiple-choice responses).

### 2.3. Procedure

For in-clinic screening, flyers were created with QR codes that linked to the mental health screeners. These flyers were hung in clinic spaces, and medical staff were trained to request that patients complete the screeners when they arrived for appointments. Patients could scan the QR codes and complete screeners on their mobile devices. As alternative options, screeners were available on iPads and on paper. All screening results were part of the patients’ medical records. This study is a retrospective chart review of patients’ medical charts from March 2021 to March 2022. IRB approval was obtained to extract relevant data for the purpose of this study. Data were extracted on 22 March 2022 and 21 March 2023.

### 2.4. Analysis

Two sets of analyses were conducted: one comparing those who were caregivers and non-caregivers, and another using the caregiving subset. The first analysis (n = 102) used the question from the caregiving youth screener, “Do you provide care for someone in your family or household who is chronically ill (lasts 3 months or more), elderly, or disabled with activities they would have difficulty doing on their own?” to differentiate the two groups. Those who responded with “Yes, I live with the person(s) who needs my help.” and “Yes, but I do not live with the person(s) who needs my help.” were grouped as caregiving youth, and those who responded “No. I don’t help anyone.” were grouped as non-caregiving youth. Using the raw score of every mental health screener, descriptive statistics were first conducted, followed by T-tests to examine the statistical significance of any differences between the caregivers and non-caregivers. Unequal variances were assumed, and the *p*-value was set at 0.05. Furthermore, a chi-squared test of independence was performed to examine the relation between caregiving and clinical cutoffs of mental health screeners, with the *p*-value also set at 0.05.

The second analysis (n = 40) was conducted with the caregiving youth subset. Using a similar process as provided by the CYP, the Level of Responsibility scale was used, based on reported types of activities performed (activities of daily living (ADLs) and instrumental activities of daily living (IADLs)) and time the caregiver spends each week performing these activities. The hours per week were given a response category (0–8 h/category 1, 9–20 h/category 2, 21–40 h/category 3, 41 or more hours/category 4) as well as the types of care (0–1 IADLs/0 ADLs/category 1, 2+ IADLs/0 ADLs/category 2, 1 ADL with or without IADLs/category 3, 2+ ADLs with or without IADLs/category 4). The combination of response categories determined the intensity of care (level 1/score 2–3, level 2/score 4, level 3/score 5, level 4/score 6–7, level 5/score 8), with levels 1 and 2 classified as low and levels 3–5 as high. T-tests were performed to compare the raw scores of the screeners to examine the statistical significance of any differences between the two groups. Unequal variances were assumed, and the *p*-value was set at 0.05.

## 3. Results

Of the 102 participants, 40 (39%) self-identified as caregiving youth. Overall, the average raw scores of all the mental health screeners for the caregiving youth (n = 40, 39%) were higher than those for the non-caregiving youth (n = 62, 61%). Compared to their non-caregiving youth counterparts, caregiving youth had statistically significant higher scores on PHQ-8 (*t* (93.76) = 2.95, *p* = 0.004), OCS (*t* (62.18) = 2.51, *p* = 0.004), and PSC-17 (*t* (85.70) = 2.92, *p* = 0.004). Furthermore, the relationship between caregiving and PHQ-8 clinical cut-off scores was significant, X^2^ (1, *N* = 102) = 3.86, *p*= 0.0495. A summary of the scores on mental health screening tools is provided in Table 1.

This subset of caregiving youth (n = 40) was 73% female (compared to 70% female of the overall sample) and ranged from 13 to 19 years of age. Caring for a parent/guardian and brother or sister were the most common care recipients, with 38% (n = 15) and 33% (n = 13) of youth caregivers responding yes to those, respectively. The other choices included great or grandparent (n = 11, 28%), another relative (n = 5, 13%), family friend (n = 1, 3%), and other (n = 3, 8%). A summary of the reported caregiving tasks is provided in Table 2. The most common caregiver tasks reported were cleaning, doing laundry (n = 20, 50%), keeping company, doing activities, reading, or playing games (n = 19, 48%), and shopping for groceries, preparing meals (n = 14, 35%). Eleven (28%) completed healthcare-related tasks such as medication management, bandaging, or managing oxygen/other medical devices. At least 10% of caregiving youth performed each of the individual tasks.

Among caregiving youth, 42.5% (n = 17) had a low Level of Responsibility (LOR), whereas 57.5% (n = 23) had a high level of responsibility. The comparison of mental health scores by LOR is presented in Table 3. There was no statistical significance between the raw scores of mental health screeners in the low-level caregiving youth and high-level caregiving youth, with the exception of a higher CAS score (*t* (18.92) = 2.22, *p* = 0.0391) among those with a lower LOR.

## 4. Discussion

Of the students surveyed, nearly 40% self-identified as caregiving youth. Although there are no comparable comparisons from the healthcare setting, this is higher than previously reported research on caregiving youth in the United States [4,5,31]. This sample differed from others in that the data were collected in a medical setting servicing high-school-aged youth (14–19 years). This high percentage may reflect the population surveyed and/or increases in caregiving by youth post-COVID. Either way, this is the first systematic screening initiative of caregiving youth in the United States in a medical setting, to our knowledge, and the high rates of endorsement justify the need for further research in this area.

Caregiving youth are engaging in a variety of tasks, similar to reports from others both in the United States and worldwide [5,7,15]. Notably, over one-quarter of the caregiving youth surveyed in this study reported providing medically related tasks, further highlighting the need for both pediatric and adult healthcare providers to be aware of this population of children. Regardless of the specific tasks, caregiving is known to be taxing [32]. A strength of this study is that validated screening tools were used to identify and measure mental health symptoms in the participants. In this cohort, caregiving was associated with more symptoms of anxiety, depression, and overall mental health concerns, including clinically significant depression, as compared to non-caregiving peers. In addition to identifying youth who are caregiving and its potential mental health effects, pediatricians, school nurses, and other healthcare providers should advocate for supportive services and legislation to help mitigate the impact of caregiving on young people.

Although the data presented are compelling, it is important to recognize the limitations of this research. First, this is a retrospective chart review study and not a prospective or comprehensive evaluation of caregiving youth. The screening measure was added to an existing clinical screening protocol to determine if there was a need to further assess this group of youth. Due to the clinical nature of this study, the number of demographic characteristics and other potential psychosocial variables of interest collected was limited. Therefore, the ability to conduct more sophisticated analyses was also limited. Future studies should consider adding a qualitative component to better understand the negative and potential positive impacts of caregiving youth experiences. It is also possible that the high prevalence of caregiving in this population was due to the sample surveyed. This was a convenience sample of youth who self-selected to seek medical services at their SBHC and may inherently have greater needs than the overall general population of the school. The SBHC was also located in a Title 1 school, indicating that the school population as a whole has higher needs and lower resources. A majority of students in the schools also identified as Black and/or Hispanic, representing groups in which it is more common to have multigenerational households [33], which could also have contributed to the higher rates of caregiving. Moreover, cultural and social expectations of the youth and families, such as the role of familism [34], were not assessed which may partially explain the expectations of youth’s role in caretaking for family members. Finally, these data were collected during the COVID-19 pandemic, which may result in skewed results. It is possible that more youth engaged in caregiving as a result of the pandemic, which may be reflected in these data. On the other hand, the youth sampled were those who continued to receive medical care during the pandemic when many chose to delay medical care at this time. It is possible that more youth are serving as caregivers in their families, but they were not receiving medical care for themselves and, therefore, did not have the opportunity to participate. Either way, it is important to recognize the potential impact of the pandemic on these data.

Further research is needed to better understand the impact of the COVID-19 pandemic on the high prevalence of caregiving identified in this population. Nonetheless, this study implies that it is feasible to add formal screening for caregiving in a pediatric clinical setting. It is important to continue to raise awareness about this population, their needs, and potential avenues for providing support among the healthcare community, so that pediatricians and other pediatric healthcare providers can actively screen for both caregiving and mental health concerns and refer adolescents for support. This can be done through a formal screening process, as in the present study, or via clinical interviews. Additional research is needed to ensure the caregiving screening tool is reliable and valid for broad-scale provider use. Regardless of the methodology, the caregiving status of youth should be documented in the electronic medical record for continued follow-up and referrals as needed.

Though mental health support for any student is available through the SBHCs and the school district [35], no specific support for or recognition of caregiving is described in the available published materials. Such services located within the schools would benefit this population, who, due to their caregiving responsibilities, may be less able to access services after school hours. In addition to traditional mental health services like individual and group therapy, there may be specific support for caregiving youth through disease-specific organizations or the Hidden Helpers Coalition for military and veteran families [36,37,38]. Informed by a 2017 study that showed a dearth of research on the impact of caregiving on children in military and veteran families, the Hidden Helpers Coalition aims to understand and support the needs of this specific population of children [38]. If formal programs are not available or accessible, caregiving youth could benefit from mentorship programs in the community, recognition of their work, insurance resources, care management, and, at minimum, supportive and nonjudgmental interactions with medical providers.

## 5. Conclusions

Screening in the healthcare system has proven effective at identifying caregiving youth as well as identifying adolescents whose mental health may be impacted by these responsibilities. Despite these high rates of caregiving youth and associated mental health concerns found in this population of caregiving youth, most pediatric healthcare providers reported being unaware of this vulnerable population in an unpublished survey [39]. There is a clear need for continued research, support, and advocacy for the caregiving youth population, but support can only be provided if youth are first identified. Implementing screening specific to caregiving youth potentially can have a meaningful impact on identifying these vulnerable youth in order to link them with supportive services.

## Figures and Tables

**Table 1:** Caregiving youth vs. non-caregiving youth scores on mental health screening tools.

Mental Health Screener	Caregiving Youth (M)	Caregiving Youth (SD)	Non-Caregiving Youth (M)	Non-Caregiving Youth (SD)	t	df	*p*
PHQ-8	9.33	4.92	6.13	5.93	2.95	93.77	0.0040 *
GAD-7	8.43	5.8	6.19	6.07	1.86	86.09	0.0661
OCS	1.3	1.59	0.58	1.08	2.51	62.18	0.0145 *
CAS	0.88	1.65	0.34	1.04	1.83	59.01	0.0719
PSC-17	13.38	6.5	9.47	6.76	2.92	85.7	0.0045 *
PSC Internalizing	5.3	2.66	3.87	3.15	2.46	92.91	0.0158 *
PSC Externalizing	3.4	2.6	2	1.98	2.91	67.48	0.0049 *

* *p* < 0.05, statistical significance. Note: M = mean. SD = standard deviation.

**Table 2:** Caregiving tasks performed by caregiving youth.

Types of Activities Performed	Number of Caregiving Youth	Percent
Cleaning, doing laundry	20	50
Keeping company, doing activities, reading, or playing games	19	48
Shopping for groceries, preparing meals	14	35
Dressing	13	33
Assisting with walking/getting in/out of a chair	12	30
Feeding	11	28
Administering medication	10	25
Bathing or showering	9	23
Translating written information or other conversations with health professionals	9	23
Providing emotional support	9	23
Organizing help from others, appointments, transportation to appointments	8	20
Taking to the bathroom or other toileting	7	18
Changing diapers or other continence care	4	10
Bandaging, assisting with oxygen or other medical equipment	4	10

**Table 3:** Mental health screening tool scores as compared by Level of Responsibility (LOR).

Mental Health Screener	Low-Level Caregiving Youth (M)	Low-Level Caregiving Youth (SD)	High-Level Caregiving Youth (M)	High-Level Caregiving Youth (SD)	t	df	*p*
PHQ-8	9.82	4.88	8.96	5.02	0.55	35.19	0.5866
GAD-7	9.06	5.23	7.96	6.27	0.61	37.38	0.5486
OCS	1.24	1.92	1.35	1.34	0.21	26.96	0.8373
CAS	1.59	2.21	0.35	0.78	2.22	18.93	0.0391 *
PSC-17	13.77	7.29	13.09	6.02	0.31	30.55	0.7565
PSC Internalizing	5.29	3.74	5.3	2.67	0.01	34.17	0.9906
PSC Externalizing	3.82	3.28	3.09	1.98	0.82	24.43	0.4192

* *p* < 0.05, statistical significance. Note: M = mean. SD = standard deviation.

## Data Availability

Requests for data can be sent to the corresponding author.
